# Fungal community profiles from Guánica State Forest caves revealed by internal transcribed spacer sequencing

**DOI:** 10.1128/mra.00817-26

**Published:** 2026-08-26

**Authors:** Francisco J. Roman-Soto, Camila M. Martínez-Llaurador, Isabel Aguirre-Ortiz, Peter A. Camacho-Nazario, Naomy M. Chaparro-Mata, Josué Rodríguez- Ramos, Ángel M. Nieves-Rivera, Miguel J. Martínez-González, Miguel Canals-Mora, Carlos Rios-Velazquez

**Affiliations:** 1 Microbial Biotechnology and Bioprospecting Laboratory, Biology Department, University of Puerto Rico 16146 , Mayagüez, Puerto Rico, USA; 2 Industrial Biotechnology Program, University of Puerto Rico 16146 , Mayagüez, USA; 3 Pacific Northwest National Laboratory, Biological Sciences Division 6865 , Richland, Washington, USA; 4 Grupo Espeleológico del Oeste, Inc. (GEO), Mayagüez, Puerto Rico, USA; Rochester Institute of Technology, Rochester, New York, USA

**Keywords:** Guánica Dry Forest, Puerto Rico, cave microbiology, taxonomic ITS profile, diversity

## Abstract

Cave ecosystems harbor fungal communities that withstand harsh conditions. This study used internal transcribed spacer sequencing to assess fungal communities across four caves in Guánica State Forest in Puerto Rico. The profiles will serve as reference, giving insights into the fungal diversity and bioprospecting within these complex and extreme ecosystems.

## ANNOUNCEMENT

Caves are extreme, oligotrophic, dark ecosystems with variable temperatures (1, 2), yet they sustain diverse microbial communities involved in nutrient cycling and the production of compounds with biomedical potential (2). Fungi contribute to decomposition, nutrient cycling, mineral weathering, and the production of antimicrobial, antioxidant, and anticancer metabolites (3, 4). Although Puerto Rican caves in the Northern Limestone Karst Belt have been characterized through 16S rDNA amplicon sequencing, and fungal communities from southern caves have been assessed using ITS sequencing, caves in Guánica State Forest remain underexplored (5, 6). Guánica Dry Forest provides a distinctive setting because subtropical dry conditions, high temperatures, seasonal water limitation, and desiccation stress may influence fungal community structure. To characterize fungal diversity and bioprospecting potential, four caves within Guánica State Forest were sampled (Table 1). Thirty grams of superficial bat guano-enriched soil was collected in sterile Whirl-Pak bags and refrigerated at 4°C until DNA extraction. Genomic DNA was extracted from 10 g of each sample suspended in 20 mL Z-buffer containing 0.02 g lysozyme. Cells were disrupted by repeated freeze–thaw cycles, followed by lysis with 2.25 mL sodium dodecyl sulfate and 1.5 mL guanidinium isothiocyanate at 65°C for 2 h. Lysates were clarified by centrifugation and extracted with an equal volume of chloroform, and the aqueous phase was precipitated with 1 vol of isopropanol at −20°C overnight. DNA pellets were centrifuged, air-dried, resuspended in T_10_E_10_ (10 mM Tris/10 mM EDTA), and stored at −20°C (7–9). DNA fragments up to 25 kb were selected by gel electrophoresis and purified with β-agarase I following the manufacturer’s protocol (10). Purified DNA was submitted to Molecular Research DNA Laboratory (MR DNA, Shallowater, TX, USA) for bTEFAP Illumina 20K ITS1–2 sequencing (11). The ITS1 region was amplified with ITS1 (5′-TCCGTAGGTGAACCTGCGG-3′) and ITS2 (5′-GCTGCGTTCTTCATCGATGC-3′) reverse primers (12) using the HotStarTaq Plus Master Mix Kit (Qiagen, USA). PCR consisted of 95°C for 5 min; 30 cycles of 95°C for 30 s, 53°C for 40 s, and 72°C for 1 min; and 72°C for 10 min. Sequencing was performed on an Illumina MiSeq using 2 × 300 bp paired-end reads. Demultiplexed reads were imported into QIIME 2 version 2026.1 in Python 3.10.14 and trimmed with ITSxpress version 2.1.4 (13). DADA2 was used for denoising without additional trimming or truncation (14). Default parameters were used unless otherwise specified. Taxonomy was assigned with the UNITE version 10.0 Dynamic Fungi Classifier released on 19 February 2025, for QIIME 2 version 2026.1. Across the four caves, more than 1.9 million non-chimeric reads were retained, with an average retention of 83.62% (Table 1). A total of 2,360 ASVs were identified. Unclassified fungi represented 18.50% of reads; Agaricales (14.37%), Mycosphaerellales (12.96%), Hypocreales (9.63%), Cantharellales (7.63%), unclassified Rozellomycota (5.99%), and Boletales (5.48%) were the most abundant groups, revealing diverse fungal communities across sites (Fig. 1).

**TABLE 1:** Sample ID and DADA2 analysis results

Sampled caves	Sample ID	Coordinates	Collection date (day/month/year)	Raw input reads	Percentage of input passed filter	Denoised	Non-chimeric	Percentage of non-chimeric
Cueva Carmen	CAR	17°57′33″ N, 66°51′10″ W	28/6/2025	653,275	97.28	564,651	573,284	81.39
Cueva Eugenio (Ño) Guerras	EUG	17°57′43″ N, 66°50′50″ W	13/10/2025	697,521	96.46	670,311	622,909	89.3
Cueva El Refugio	REF	17°57′35″ N, 66°51′11″ W	28/6/2025	441,982	97.42	428,770	357,405	80.86
Sumidero Lluveras	SUL	17°57′51″ N, 66°50′45″ W	13/10/2025	433,895	93.76	405,038	359,723	82.91

**Fig 1:**
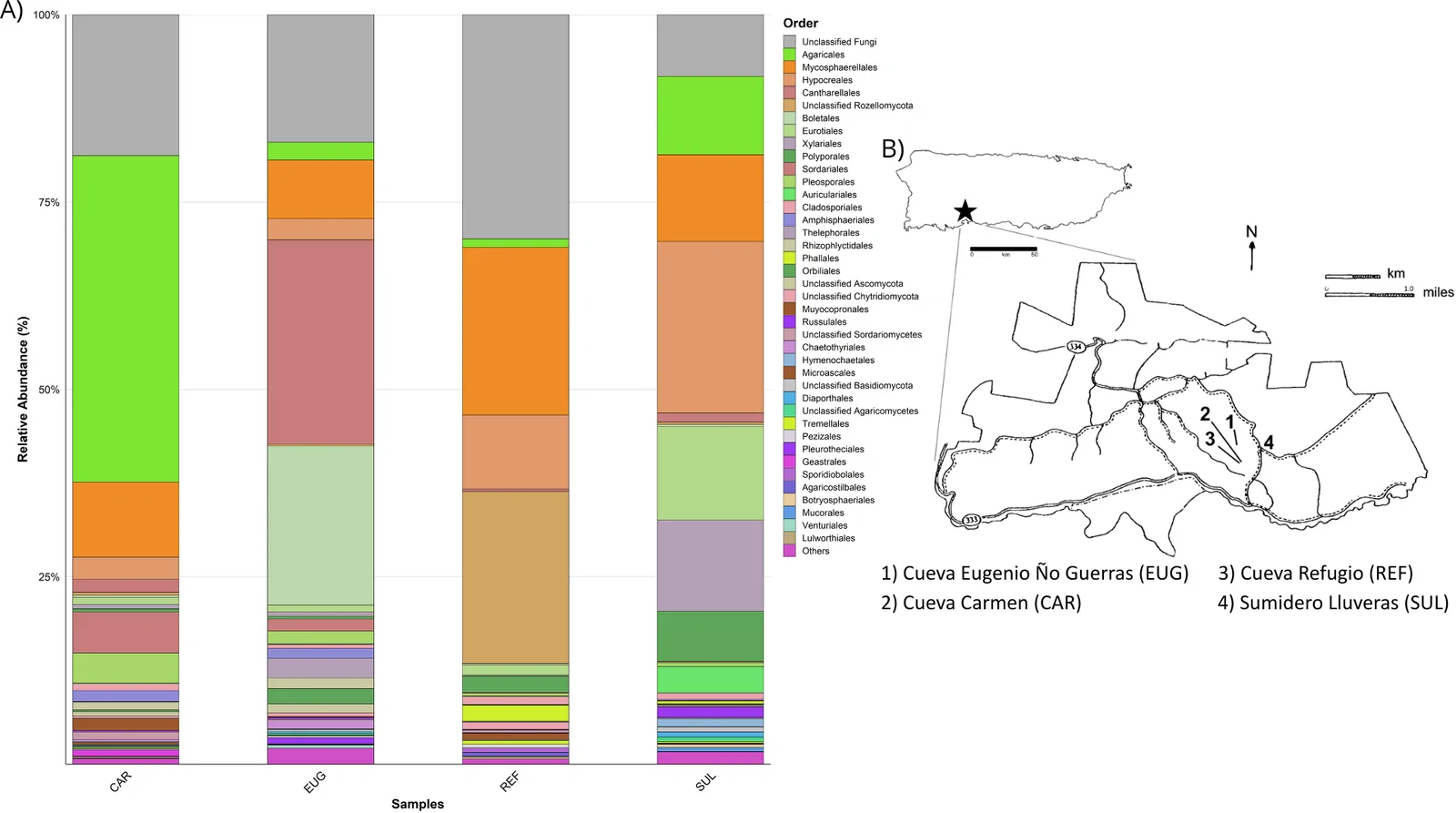
Relative abundance of fungal communities at the order level across four Puerto Rican caves. (**A**) Taxa bar plots showing the relative abundance of fungal orders in each cave. (**B**) Map of Guánica State Forest indicating the locations of the sampled caves. The map was generated by Ángel M. Nieves-Rivera.

## Data Availability

The raw data from this project has been deposited in the National Center for Biotechnology Information under BioProject accession number PRJNA1457880. The samples were distributed as follows: Cueva Carmen (CAR) (SRA accession number SRX33109461), Cueva Eugenio No Guerra (EUG) (SRA accession number SRX33109462), Cueva El Refugio (REF) (SRA accession number SRX33109463), and Sumidero Lluveras (SUL) (SRA accession number SRX33109464).
